# *MAL62* overexpression and *NTH1* deletion enhance the freezing tolerance and fermentation capacity of the baker’s yeast in lean dough

**DOI:** 10.1186/s12934-016-0453-3

**Published:** 2016-04-04

**Authors:** Xi Sun, Cui-Ying Zhang, Ming-Yue Wu, Zhi-Hua Fan, Shan-Na Liu, Wen-Bi Zhu, Dong-Guang Xiao

**Affiliations:** College of Biological Engineering, Tianjin Agricultural University, Tianjin, 300384 People’s Republic of China; Tianjin Engineering Research Center of Agricultural Products Processing, Tianjin, 300384 People’s Republic of China; Key Laboratory of Industrial Fermentation Microbiology, Ministry of Education, Tianjin Industrial Microbiology Key Laboratory, Tianjin University of Science and Technology, Tianjin, 300457 People’s Republic of China; College of Biotechnology, Tianjin University of Science and Technology, Tianjin, 300457 People’s Republic of China; Diagreat Biotechnologies., Ltd, Beijing, 101111 People’s Republic of China

**Keywords:** Baker’s yeast, *MAL62*, *NTH1*, Freezing tolerance, Cell viability, Leavening ability

## Abstract

**Background:**

Trehalose is related to several types of stress responses, especially freezing response in baker’s yeast (*Saccharomyces cerevisiae*). It is desirable to manipulate trehalose-related genes to create yeast strains that better tolerate freezing-thaw stress with improved fermentation capacity, which are in high demand in the baking industry.

**Results:**

The strain overexpressing *MAL62* gene showed increased trehalose content and cell viability after prefermention-freezing and long-term frozen. Deletion of *NTH1* in combination of *MAL62* overexpression further strengthens freezing tolerance and improves the leavening ability after freezing-thaw stress.

**Conclusions:**

The mutants of the industrial baker’s yeast with enhanced freezing tolerance and leavening ability in lean dough were developed by genetic engineering. These strains had excellent potential industrial applications.

## Background

Frozen dough technology has been used in bakery industry to provide consumers with high-quality fresh bakery and convenience. However, cellular macromolecules, including proteins, nucleic acids and lipids of the yeast used in frozen dough, could be seriously damaged under the freezing and the subsequent thawing treatments, leading to inhibition of cell growth, cell viability and the leavening ability [1].

A great body of knowledge is already available regarding the molecular responses of the baker’s yeast (*Saccharomyces cerevisiae*) to frozen dough-associated stresses [2]. Among other molecules, trehalose has been highlighted due to its main function as a protective molecule in stress response [3]. This effect is achieved either by protecting membrane integrity through the union with phospholipids [4], or by preserving the native conformation of proteins and preventing aggregation of partially denatured proteins [5].

When yeast cells suffer from freezing stress, they accumulate large amounts of trehalose [6]. The accumulation is mainly induced by the classical the UDPG-dependent trehalose synthesis pathway, or referred as system I. It contains a trehalose-6-phosphate synthase encoded by *TPS1* [7], a trehalose-6-phosphate phosphatase encoded by *TPS2* [8] and a trehalose-synthesis protein complex encoded by *TSL1* [9]. In addition, an alternative trehalose synthesis pathway, called ADPG-dependent trehalose synthesis pathway or the system II, has been proposed [10, 11]. It is specifically linked to maltose utilization.

Maltose metabolism in yeast depends on at least one of the five unlinked *MAL* loci (*MAL1* through *MAL4* and *MAL6*). A typical *MAL* locus consists of a *MALx1* (*MALxT*) gene (where *x* is the locus), encoding maltose permease, a *MALx2* (*MALxS*) gene, coding for alpha-glucosidase (maltase), and a *MALx3* (*MALxR*) gene, encoding a positive regulatory protein [9]. It is reported that the expression of any one of the *MAL* loci in *MAL*-constitutive strains could elicit a maltose-induced trehalose accumulation [11]. Studies have shown that maltose and trehalose seem to share a common regulating mechanism [17, 18]. The maltose permease has been considered the rate-limiting enzyme in the MAL genes induction and maltose metabolism [4, 6, 12]. Hence, attempts to increase the trehalose content by system II had been concentrated on the modification of maltose permease or the entire MAL gene cluster [19–21]. However, recent studies showed that the alpha-glucosidase (maltase) is more important than maltose permease in maltose metabolism and leavening ability of baker’s yeast in lean dough [22–24]. In addition, the system II might be dependent of the system I, due to the fact that the system II is completely prevented when *TPS1*, a key gene in system I, is deleted [12].

Trehalose degradation could also be induced under certain stress [13, 14]. The best characterized trehalase is the neutral trehalase encoded by the *NTH1* gene, which is induced by stress, such as heat. Nth1p is involved in thermos-tolerance and hydrolyzes intracellular trehalose into glucose [15, 16]. Deletion of *NTH1* results in accumulation of trehalose, and heat sensitivity.

To better understand the role of trehalose in freezing tolerance of baker’s yeast in lean dough, and its possible mechanism, we investigated the effects of overexpression of *MAL62,* the gene encoding an alpha glucosidase, and deletion of *NTH1* gene, on trehalose accumulation and on the freezing tolerance and leavening ability of baker’s yeast in lean dough.

## Methods

### Strains, plasmids and growth conditions

The genetic properties of all *S. cerevisiae* strains and plasmids used in the present study are summarized in Table 1. The BY14a was selected as a high leavening capacity haploid from 32 clones derived from the diploid BY14 strain, which has been maintained at the Tianjin Key Laboratory of Industrial Microbiology, Tianjin University of Science and Technology.

**Table 1:** Characteristics of strains and plasmids used in the present study

Strains or plasmids	Relevant characteristic	Reference or source
Strains		
Escherichia coli DH5α	Φ80 *lacZ*ΔM15 *ΔlacU169 recA1 endA1 hsdR17 supE44 thi*-*1 gyrA relA1*	Yeast Collection Center of the Tianjin Key Laboratory of Industrial Microbiology
BY14	*MATa/a* Industrial baker’s yeast	Yeast Collection Center of the Tianjin Key Laboratory of Industrial Microbiology
BY14a^a^	*MATa*, haploid derived from BY14 strain	This study
B-NTH1	*MATa*, Δ*NTH1*:: *loxP*	This study
B-NTH1 + K	*MATa*, Δ*NTH1*:: *loxP*, Yep-K	This study
BY14a + K	*MATa*, Yep-K	This study
B + MAL62	*MATa*, Yep-PMK	This study
B-NTH1 + MAL62	MATa, Δ*NTH1*:: *loxP*, Yep-PMK	This study
Plasmids		
pUG6	*E. coli/S. cerevisiae* shuttle vector, containing *Amp* ^+^, *loxP*-*kanMX*-*loxP* disruption cassette	[41]
Yep352	*URA3* ^+^, *Amp* ^*R*^ *ori* control vector	Invitrogen, Carlsbad, Ca, USA
Yep-K	*KanMX ARS URA3* ^+^, *Amp* ^*R*^ *ori* control vector	This study
pPGK1	*bla LEU2 PGK1*_*P*_-*PGK1*_*T*_	[42]
pPGKM	*bla LEU2 PGK1*_*P*_-*MAL62*-*PGK1*_*T*_	This study
pUC-ABK	NA-*loxp*-*KanMX*-*loxp*-NB	Yeast Collection Center of the Tianjin Key Laboratory of Industrial Microbiology
Yep-PMK	*bla LEU2 PGK1*_*P*_-*MAL62*-*PGK1*_*T*_ *, KanMX*	This study
pSH-Zeocin	*Zeo* ^*r*^, *Cre* expression vector	Yeast Collection Center of the Tianjin Key Laboratory of Industrial Microbiology

^a^BY14a was selected as high leavening capacity haploid from 32 clones derived from BY14 strain (data not shown)

Recombinant DNA was amplified in *Escherichia coli DH5a*. Transformants were grown in Luria–Bertani medium (10 g/L tryptone, 5 g/L yeast extract, and 10 g/L NaCl) with 100 mg/L ampicillin. The plasmid was obtained using Plasmid Mini Kit II (D6945, Omega, USA).

The yeast strain was grown at 30 °C in yeast extract peptone dextrose (YEPD) medium (10 g/L yeast extract, 20 g/L peptone, and 20 g/L dextrose). Approximately 800 mg/L of G418 was added to the YEPD plates for selecting Geneticin (G418)-resistant transformants. After cultivation in YEPD for 24 h, 20 mL of the cell culture was inoculated into 200 mL of cane molasses medium (5 g/L yeast extract, 0.5 g/L (NH_4_)_2_SO_4_, and 12° Brix cane molasses) at the initial OD_600_ = 0.4 and cultivated for 24 h at 30 °C with 180 rpm rotary shaking to the final OD_600_ = 1.8. Cells were harvested through centrifugation (4 °C, 1500×*g*, 5 min) and were washed twice with sterile water at 4 °C for the succeeding fermentation experiments. To investigate the degradation of trehalose during prefermentation and the freezing tolerance, a modified the low sugar model liquid dough (LSMLD) medium was used [17]. The modified medium contains 2.5 g/L (NH_4_)_2_SO_4_, 5 g/L urea, 16 g/L KH_2_PO_6_, 5 g/L Na_2_HPO_4_, 0.6 g/L MgSO_4_, 22.5 mg/L nicotinic acid, 5 mg/L Ca-pantothenate, 2.5 mg/L thiamine, 1.25 g/L pyridoxine, 1 mg/L riboflavin, and 0.5 mg/L folic acid and carbon sources (33.25 g/L maltose with 5 g/L glucose).

### Plasmid construction and yeast transformation

Genomic yeast DNA was prepared from the industrial baker’s yeast BY14a using a yeast DNA kit (D3370-01, Omega, Norcross, GA, USA). Table 2 shows the PCR primers used in this study.

**Table 2:** Primers used in the present study (restriction sites are underlined)

Primer name	Sequence 5′-3′
Kan-U	CGGGGTACCCAGCTGAAGCTTCGTACGC
Kan-D	CGCGGATCCGCATAGGCCACTAGTGGATCTG
MAL62-U	CCGCTCGAGATGACTATTTCTGATCATCC
MAL62-D	CCGCTCGAGTTATTTGACGAGGTAGATT
PGK-U	CGCGGATCCAAGCTTTCTAACTGATCTATCCAAAACTGA
PGK-D	CGCGGATCCAAGCTTTAACGAACGCAGAATTTTC
N-S	ATCATCATCTGTAATCGCTTCACC
K-S	CCTTTTATATTTCTCTACAGGGGCG
N-X	TACAGCGGTAAAGTTTCTATGAGCA
K-X	TAGGTTGTATTGATGTTGGACGAGT

Plasmid Yep-PMK (Yep-*PGK1*-*MAL62*-*KanMX*), an episomal plasmid with *MAL62* under the control of the constitutive yeast phosphoglycerate Kinase gene (*PGK1*) promoter (*PGK1*_*P*_) and terminator (*PGK1*_*T*_), was constructed as follows: a *Kpn*I/*BamH*I*KanMX* fragment, which was the dominant selection marker during yeast conversion, was amplified through PCR using pUG6 as template with Kan-U and Kan-D primers, and was cloned to the Yep352 vector to construct the empty plasmid Yep-K (Yep-KanMX). A *Xho*I fragment of *MAL62* amplified with MAL62-U and MAL62-D primers from the genomes of the parental strain BY14a was inserted into the *PGK1* fragment of pPGK1 vector and resulted in plasmid pPGKM. Then, the *BamH*I fragment of PGKM (the entire *PGK1* and the inserted *MAL62*) amplified with PGK-U and PGK-D from pPGKM was cloned to Yep-K to produce the final plasmid Yep-PMK.

Baker’s yeast transformation was achieved through lithium acetate/PEG method [18]. The deletion cassette of NA-*loxP*-*KanMX*-*loxP*-NB was amplified with N-S and N-X and transformed into the industrial baker’s yeast BY14a. The fragment was integrated into the chromosome at the *NTH1* locus of BY14a by homologous recombination to construct the *NTH1* deletion strain. The selection of *NTH1* deletion strain was performed using the YEPD medium supplemented with 800 mg/L geneticin (G418). After selection, recombinant strains were verified with the primers N-S, K-S and N-X, K-X. Cre recombinase was expressed and *KanMX* was excised after introducing the plasmid pSH-Zeocin into the *NTH1* deletion strain, thus resulting in B-NTH1. The respective transformation plasmids Yep-K, Yep-PMK were then transformed to select the G418-resistant strains BY14a + K, B-NTH1 + K, B + MAL62 and B-NTH1 + MAL62. BY14a + K and B-NTH1 + K were BY14a and B-NTH1 carrying the vector Yep-K, respectively, used as a blank control to demonstrate any possible effect of the empty vector. The transformants were then verified by PCR using the primers Kan-U and Kan-D.

### Assay of the intracellular trehalose content

Fresh yeast cells were dried overnight at 85 °C to calculate the cell dry weight (CDW). Trehalose was extracted from 0.1 g of fresh yeast cells (previously washed with distilled water twice) with 4 mL of 0.5 mol/L cold trichloroacetic acid and the extract was employed for measuring the trehalose content as described previously [19, 20]. Experiments were conducted three times.

### Determination of neutral trehalase activity

The activities of neutral trehalase in crude extracts were measured as described previously [21]. The liberated glucose was analyzed by HPLC employing an Aminex HPX-87H column with 5 mmol/L H_2_SO_4_ as the mobile phase at a flow rate of 0.6 mL/min at 65 °C. One unit of trehalase activity was defined as the amount of trehalase producing 1.0 μm glucose per min under assay conditions. The specific trehalase activity was expressed as the units per gram CDW. Experiments were conducted three times.

### Determination of Tps1 (trehalose-6-phosphate synthase) activity

Tps1 activity was measured as described previously [22]. The trehalose-6-phosphate formed during the reaction was quantitatively determined using the Anthrone method [19]. One unit of Tps1 activity was defined as the amount of Tps1 producing 1.0 μm 6-phosphate-trehalose per min under assay conditions. The specific Tps1 activity was expressed as the units per gram CDW. Experiments were conducted three times.

### Determination of alpha-glucosidase activity

Crude extracts were prepared using the Salema-Oom method to determine enzyme activities [23]. Alpha-glucosidase were determined following the Houghton-Larsen method [24]. Standard errors were less than 10 %.

### Determination of the cell viability of baker’s yeast after freezing and thaw

For the freeze–thaw stress, yeast cells were harvested from the cane molasses medium and inoculated into the LSMLD medium at 30 °C for 25 min. One milliliter of cell culture was shifted to −20 °C and at 5 min intervals for different prefermentation time periods. After freezing for 1–3 week, the frozen suspensions were thawed at 30 °C for 30 min then diluted and plated on YEPD plates for 2 days. Cell viability was determined by the percentage of the number of colonies after stressing relative to the number of colonies before stress. Three independent experiments were performed.

### Determination of leavening ability

The leavening ability of yeast cells was assayed by measuring the CO_2_ production in lean dough. Lean dough was composed of 280 g of standard flour, 150 mL of water, 4 g of salt, and 9 g of fresh yeast. The dough was evenly and rapidly stirred for 5 min at 30 ± 0.2 °C then divided into pieces (50 g each) and placed in a fermentograph box 171 (Type JM451, Sweden). CO_2_ production was recorded at 30 °C for 120 min. Experiments were conducted three times.

To assay the leavening ability after freeze–thaw, the mixed dough was stored at −20 °C. After freezing for 1 week, the frozen dough was thawed at 30 °C for 30 min, and the CO_2_ production was assayed for 120 min at 30 °C. Experiments were conducted at least thrice.

### Statistical analysis

Data were expressed as mean ± SD and were accompanied by the number of experiments independently performed. Differences among all the strains were analyzed using ANOVA. *P* < 0.05 were considered statistically significant. The differences between the transformants and the parental strain were confirmed by Student’s *t* test. Differences at *P* < 0.05 were considered statistically significant.

## Results

### Overexpression of *MAL62* enhances the Tps1 activity and intracellular trehalose content of baker’s yeast

Previous studies have reported that the *MAL* gene has a positive effect on the activity of Tps1, a trehalose-6-phosphate synthase that synthesizes trehalose under stress conditions [12]. We first tested if the Tps1 activity is affected by *MAL62* overexpression. As shown in Table 3, overexpression of *MAL62* (in both B + MAL62 and B-NTH1 + MAL62) significantly increased the Tps1 activity (*P* < 0.05). The alpha-glucosidase activities of these two strains were also increased significantly (Table 3). These results suggest that overexpression of *MAL62* induces trehalose production.

**Table 3:** Alpha-glucosidase activities, Tps1 activities and the neutral trehalase activities of strains

	Alpha-glucosidase activity^a^ (μmol/mg/min)	Tps1 activity^a^ (U/g CDW)	Neutral trehalase activity^b^ (U/g CDW)
BY14a	2.46 ± 0.25	0.80 ± 0.07	12.28 ± 0.88
BY14a + K	2.45 ± 0.22	0.83 ± 0.10	12.26 ± 0.81
B-NTH1	2.45 ± 0.21	0.82 ± 0.07	8.68 ± 0.74*
B-NTH1 + K	2.45 ± 0.23	0.81 ± 0.09	8.39 ± 0.53*
B + MAL62	4.43 ± 0.37**	1.06 ± 0.10*	12.36 ± 0.93
B-NTH1 + MAL62	3.66 ± 0.32**	1.01 ± 0.11*	8.31 ± 0.61*

Values shown represent at least three independent experiments (data are mean ± SD). Significant difference of the transformants (BY14a + K, B-NTH1,B-NTH1 + K, B + MAL62, B-NTH1 + MAL62) from the parental strain was confirmed by Student’s *t*-test (**P < 0.01,*P < 0.05, n = 3)

^a^Alpha-glucosidase activities and Tps1 activities were calculated from the cells grown in cane molasses medium

^b^Neutral trehalase activities were calculated from the cells prefermentation in LSMLD medium

**Fig. 1:**
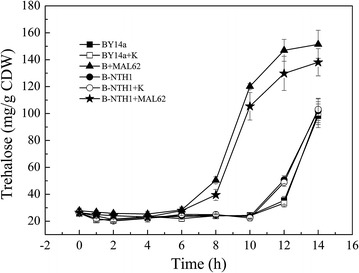
Trehalose accumulation during growth of the six *S. cerevisiae* strains in cane molasses medium. BY14a + K and B-NTH1 + K were BY14a and B-NTH1 carrying the vector Yep-K, respectively, used as a blank control to demonstrate any possible effect of the empty vector. Data are average of three independent experiments, and *error bars* represent ± SD

To further confirm this, we measured and compared the trehalose levels in different strains. We found that all six strains (BY14a, B-NTH1, B + MAL62, B-NTH1 + MAL62, BY14a + K and B-NTH1 + K) had similar growth curves. Cells entered exponential phase 3 h after inoculation, and stationary phase 10 h after inoculation (data not shown). Our results showed in strains overexpressing *MAL62* (B + MAL62 and B-NTH1 + MAL62), trehalose started to accumulate in late exponential stage at a rate of 21.9 mg/h/g CDW. In contrast, in strains having no *MAL62* overexpression (BY14a, B-NTH1, BY14a + K and B-NTH1 + K), trehalose accumulation started only in stationary phase and at a lower rate (19.1 mg/h/g CDW) (Fig. 1).

### *MAL62* overexpression does not affect the rate of trehalose degradation

**Fig. 2:**
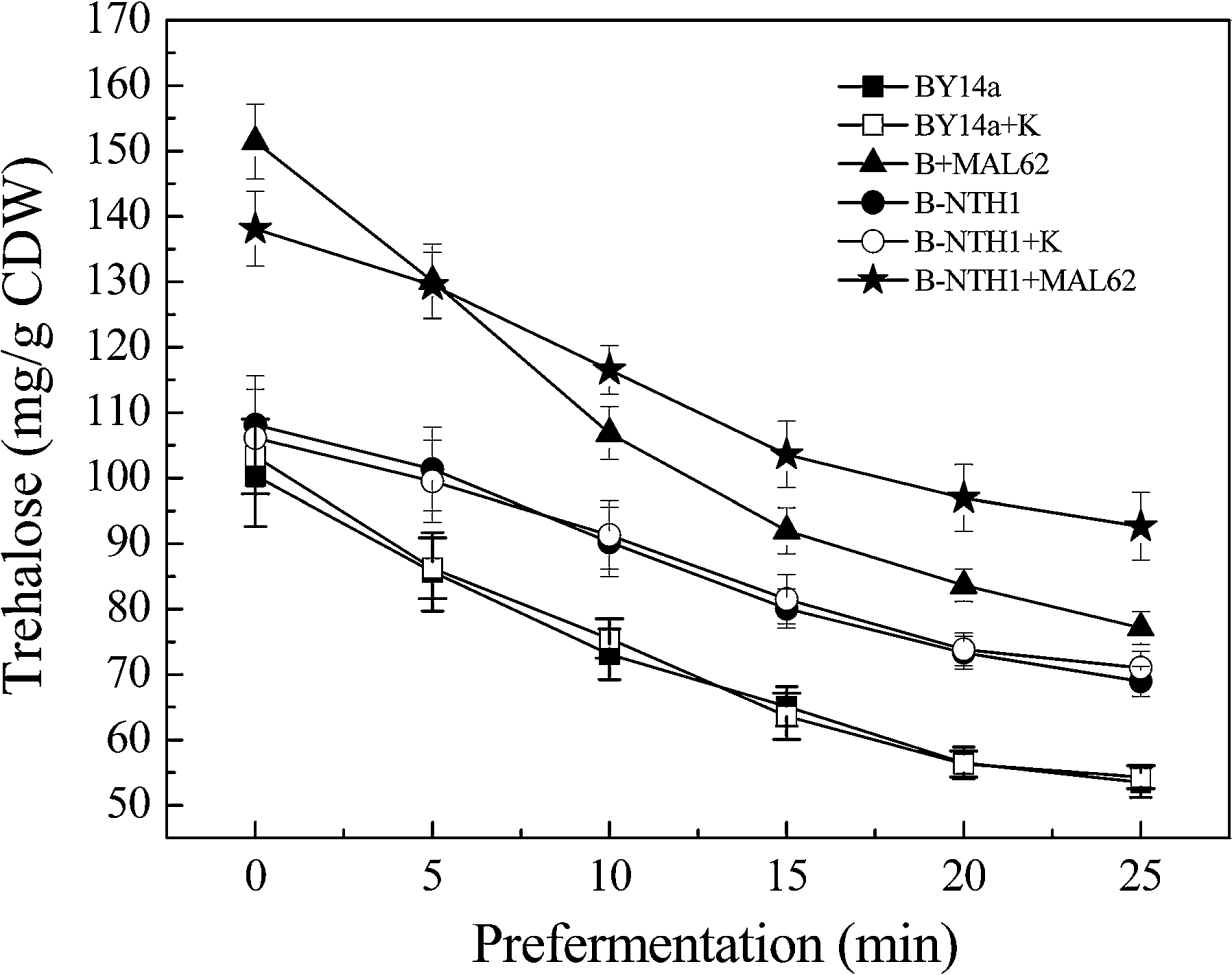
Content of intracellular trehalose during cultivation in LSMLD (prefermentation). Data are averages of three independent experiments, and *error bars* represent ± SD

To examine if *MAL62* overexpression or *NTH1* is involved in trehalose degradation, we compared the neutral trehalase activity and the degradation rate of intracellular trehalose among the six strains. As shown in Table 3, the B + MAL62 strain had a similar neutral trehalase activity compared to its control (BY14a and BY14a + K), suggesting that overexpression of *MAL62* did not affect the trehalose degradation. This is further confirmed by direct measurement of the intracellular trehalose content (Fig. 2), which showed a similar degradation rate among B + MAL62, BY14a and BY14a + K. In addition, both the neutral trehalase activity and the rate of trehalose degradation were significantly lower in all *NTH1* deletion strains (B-NTH1, B-NTH1 + K and B-NTH1 + MAL62) (Table 3, Fig. 2), regardless whether *MAL62* was overexpressed or not. These results suggest that *NTH1*, but not *MAL62*, is important for trehalose degradation.

### High trehalose content increases viability of yeast cells after freezing

Although a number of reports have shown that the degradation of trehalose during prefermentation is necessary [25], the residual intracellular trehalose is still considered to be important to freezing tolerance of yeast [26, 27]. Hereby, we assessed the cell viability of the six strains to investigate the effect of *MAL62* overexpression and/or *NTH1* deletion on the freezing tolerance of yeasts after prefermentation and 7 d freezing.

**Fig. 3:**
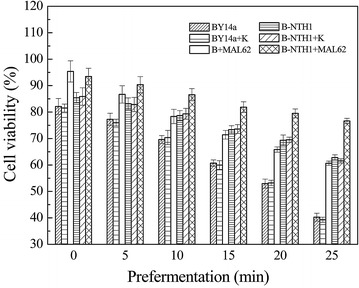
Cell viability of strains after prefermentation for different time periods in LSMLD and frozen for 7 d at −20 °C. Data are average of three independent experiments, and *error bars* represent ± SD

As shown in Fig. 3, the cell viability of strains with *MAL62* overexpression (B + MAL62 and B-NTH1 + MAL62) was significantly higher than the other strains before prepermentation (time = 0 min). Cell viability of all strains decreased as prefermentation time increased. 25 min after prefermentation, the cell viability of the strain with both *MAL62* overexpression and *NTH1* deletion (B-NTH1 + MAL62) was significantly higher than other strains (ANOVA, *P* < 0.05). The cell viability of strains with either *MAL62* overexpression or *NTH1* deletion remained in the middle, while the control strains (BY14a and BY14a + K) had the lowest viability, dropping from about 80 % to about 40 %. The cell viability is in agreement with the trehalose content (Fig. 2), which showed that 25 min after prefermentation, the B-NTH1 + MAL62 had the highest trehalose content (95 mg/g CDW) and the controls (BY14a and BY14a + K) had the lowest (about 55 mg/g CDW). These results suggest that the residual trehalose content has a positive correlation with the viability of yeast cells after prefermentation and freezing [28].

### Overexpression of *MAL62* or deletion of *NTH1* confers long-term freezing tolerance of baker’s yeast

**Fig. 4:**
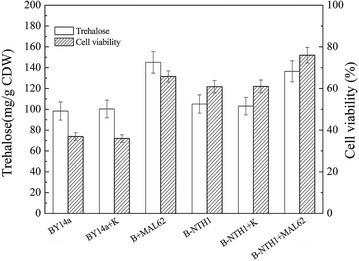
Intracellular trehalose content (before frozen) and cell viability (after frozen) of strains before or after 21d frozen. Data are average of three independent experiments, and *error bars* represent ± SD

In order to access the long-term freezing tolerance of the *NTH1*-deletion and/or the *MAL62*-overexpression strains, we examined the trehalose content before freezing and the cell viability 21d after freezing (Fig. 4). As shown in Fig. 4, both the trehalose content and the cell viability were significantly higher in strains with *MAL62* overexpression (B + MAL62 and B-NTH1 + MAL62) (ANOVA, *P* < 0.05). Compared with the control (BY14a and BY14a + K), deletion of *NTH1* alone (B-NTH1 and B-NTH1 + K) also induced a higher trehalose content and higher cell viability, which is in agreement with previous studies [26, 29].

### Overexpression of *MAL62* and deletion of *NTH1* enhance the fermentation characteristics of baker’s yeast exposed to freezing-thaw stress

**Fig. 5:**
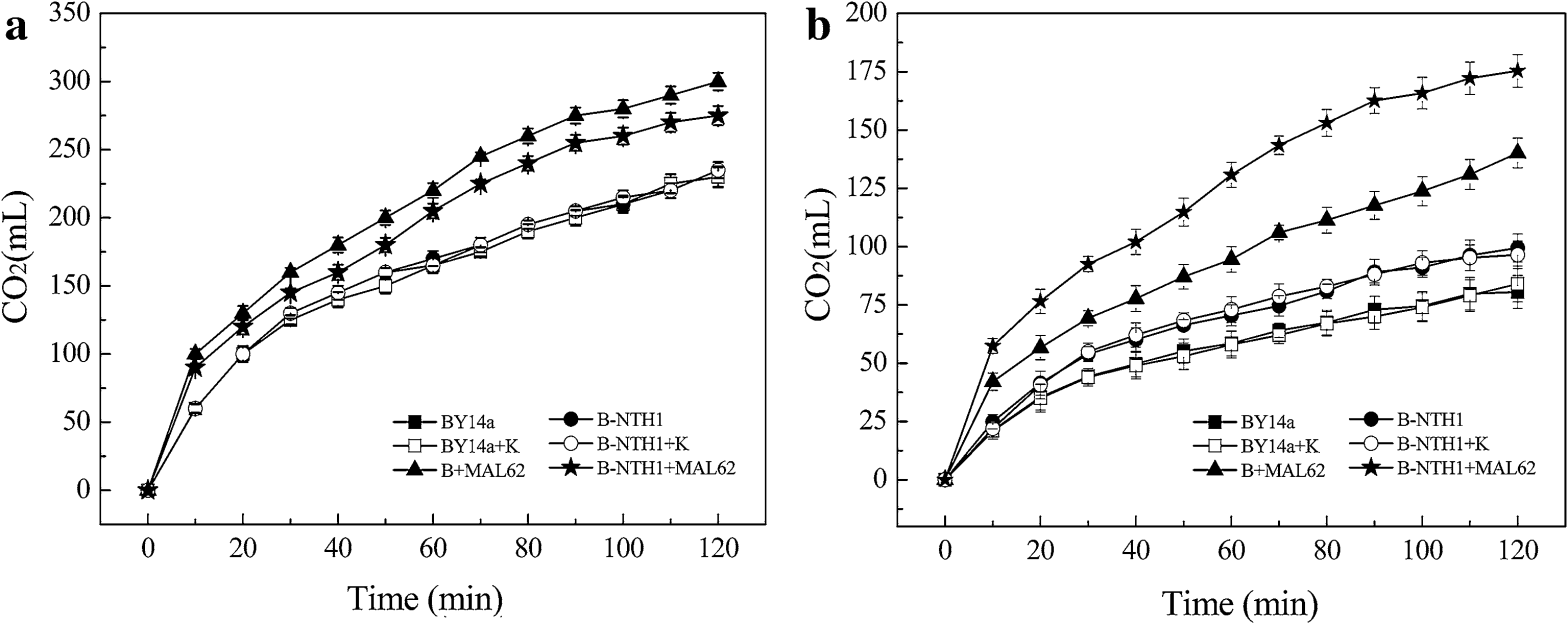
CO_2_ production of yeast in lean dough before and after freeze–thaw. CO_2_ production was measured before (**a**) and after (**b**) freeze–thaw stress. Data are average of three independent experiments, and *error bars* represent ± SD

Leavening ability is an important fermentation characteristic of baker’s yeast used in frozen dough. We next explored the possible effects of *MAL62* overexpression and *NTH1* deletion on the leavening ability after freezing and thaw by measuring the CO_2_ production. Our results showed that freezing-thaw caused a reduction of CO_2_ production in all strains (comparing Fig. 5a with 5b). However, either before or after freezing-thaw, overexpression of *MAL62* (B + MAL62 and B-NTH1 + MAL62) significantly enhanced the CO_2_ production (ANOVA, *P* < 0.05). *NTH1* deletion alone had no effect on CO_2_ production before freezing-thaw (Fig. 5a) but enhanced the CO_2_ production after freezing-thaw (Fig. 5b). Interesting, *MAL62* overexpression and *NTH1* deletion (B-NTH1 + MAL62) had a lower CO_2_ production than *MAL62* overexpression alone (B + MAL62) before freezing-thaw, but the CO_2_ production was higher after the freezing-thaw, suggesting that *MAL62* overexpression and *NTH1* deletion provide the best enhancement on leavening ability upon freezing-thaw stress.

## Discussion

Biological macromolecules and membranes are liable to denaturation under freezing conditions [30], Freezing also causes the formation of intracellular ice crystals, which are harmful to cells. It has been suggested that trehalose could act as a stabilizer of cellular membranes and proteins under freezing stress [28]. Previous studies have reported that the modification of the whole *MAL* gene cluster is necessary to elicit trehalose synthesis [31, 32]. In this study, we demonstrated that the single-gene-overexpression of *MAL62* in industrial baker’s yeast is capable of increasing trehalose accumulation and cell viability under freezing stress. Trehalose formation in *MAL62* overexpressing strains (B + MAL62 and B-NTH1 + MAL62) was earlier and faster than the controls (Fig. 1), suggesting the positive effects on the intracellular trehalose content and freezing tolerance (Figs. 2, 4). Moreover, although *MAL62* overexpression had little effect on protecting trehalose against degradation during prefermentation, the cell viability assay showed that the *MAL62* overexpression could protect cells against freezing stress after prefermentation. This is in line with a previous report [33], showing that the trehalose accumulation before the induction of stress was more important for stress tolerance.

One explanation is that *MAL62* overexpression enhances the activity of Tps1. This hypothesis relies on the fact that maltose constitutive genes could partially relieve Tps1 from the catabolite repression [34], and the alpha-glucosidase (coded by gene *MAL62*) is the rate-limiting factor in maltose metabolism [35]. Our result is consistent with this hypothesis, since Tps1 activity could increase when the alpha-glucosidase activity was enhanced by *MAL62* overexpression (Table 3). Another explanation is that the existence of adenosine-diphosphoglucose (ADPG)-dependent trehalose synthase, which requires ADPG instead of UDPG as donor of glucose units for trehalose synthesis [36]. Since the expression of ADPG-pyrophosphorylase gene and *MAL* genes shared the common regulation, any of the *MAL* gene products either by means of control at the transcription level, or by acting directly on enzyme activity could regulate the activity of the ADPG-pyrophosphorylase activity [11]. Hence, overexpression of *MAL62* alone could increase intracellular trehalose content and bring about further enhancements in freezing tolerance.

The fermentation characteristics of baker’s yeast as a strong correlation with the tolerance in stress conditions [37]. After exposure to freeze–thaw stress, response to the environmental change involved in rapid accumulation of relevant protectants and rapid production of enzymes related to stress-protective effect [7, 19]. In this work, we found that the freezing tolerance and the fermentation characteristics of the double mutant (B-NTH1 + MAL62) were significantly enhanced than that of either single mutant (B-NTH1 or B + MAL62) after the freezing-thaw stress (Figs. 4, 5b). In addition, we found that *NHT1* deletion (B-NTH1, B-NTH1 + K and B-NTH1 + MAL62) induced a low neutral trehalase (Table 3), which caused a lower level of trehalose degradation. High activity of trehalose synthase (+MAL62) [38] and low activity of neutral trehalase (−NTH1) increase the intracellular trehalose level [26], which explains why the double mutant (B-NTH1 + MAL62) provides the best freezing tolerance and fermentation characteristics [39].

In summary, our study showed that *MAL62* overexpression and *NTH1* deletion in baker’s yeast significantly enhanced the freezing tolerance and fermentation characteristics, which is in high demand in the frozen dough baking industry [40]. On the basis of our findings, it is also possible to lengthen the storage period of frozen dough through overexpression of the *MAL62* with *NTH1* deletion. It provides valuable insights for breeding novel stress-tolerant and fast-fermented baker’s yeast strains that are useful for baking industry.

## Conclusion

The results of this study show that overexpression of *MAL62* was an effective way of increasing trehalose content and cell viability after prefermention-freezing and long-term frozen. Deletion of *NTH1* in combination of *MAL62* overexpression could further strengthen freezing tolerance and improve the leavening ability after freezing-thaw stress. Furthermore, the single-gene-overexpression of *MAL62* in industrial baker’s yeast is capable of increasing trehalose accumulation, therefore, promoting cell viability and the leavening ability of baker’s yeast in lean dough under freezing stress. Hence, such baker’s yeast has excellent commercial and industrial applications.
